# Outbreaks of *Streptococcus pneumoniae *carriage in day care cohorts in Finland – implications for elimination of transmission

**DOI:** 10.1186/1471-2334-9-102

**Published:** 2009-06-27

**Authors:** Fabian Hoti, Panu Erästö, Tuija Leino, Kari Auranen

**Affiliations:** 1 Department of Vaccination and Immune Protection, Division of Health Protection, National Institute for Health and Welfare (THL), Helsinki, Finland

## Abstract

**Background:**

Day care centre (DCC) attendees play a central role in maintaining the circulation of *Streptococcus pneumoniae *(pneumococcus) in the population. Exposure within families and within DCCs are the main risk factors for colonisation with pneumococcal serotypes in DCC attendees.

**Methods:**

Transmission of serotype specific carriage was analysed with a continuous time event history model, based on longitudinal data from day care attendees and their family members. Rates of acquisition, conditional on exposure, were estimated in a Bayesian framework utilising latent processes of carriage. To ensure a correct level of exposure, non-participating day care attendees and their family members were included in the analysis. Posterior predictive simulations were used to quantify transmission patterns within day care cohorts, to estimate the basic reproduction number for pneumococcal carriage in a population of day care cohorts, and to assess the critical vaccine efficacy against carriage to eliminate pneumococcal transmission.

**Results:**

The model, validated by posterior predictive sampling, was successful in capturing the strong temporal clustering of pneumococcal serotypes in the day care cohorts. In average 2.7 new outbreaks of pneumococcal carriage initiate in a day care cohort each month. While 39% of outbreaks were of size one, the mean outbreak size was 7.6 individuals and the mean length of an outbreak was 2.8 months. The role of families in creating and maintaining transmission was minimal, as only 10% of acquisitions in day care attendees were from family members. Considering a population of day care cohorts, a child-to-child basic reproduction number was estimated as 1.4 and the critical vaccine efficacy against acquisition of carriage as 0.3.

**Conclusion:**

Pneumococcal transmission occurs in serotype specific outbreaks of carriage, driven by within-day-care transmission and between-serotype competition. An amplifying effect of the day care cohorts enhances the spread of pneumococcal serotypes within the population. The effect of vaccination, in addition to reducing susceptibility to pneumococcal carriage in the vaccinated, induces a herd effect, thus creating a counter-effect to the amplifying effect of the cohort. Consequently, the critical vaccine efficacy against carriage, required for elimination of transmission, is relatively low. Use of pneumococcal conjugate vaccines is expected to induce a notable herd protection against pneumococcal disease.

## Background

Knowing transmission is key to understanding vaccine prevention of diseases caused by the pneumococcus (*Streptococcus pneumoniae*). The adoption of new pneumococcal polysaccharide conjugate vaccines into national vaccination programs has been indicated by their efficacy in protecting the vaccinated individuals against invasive pneumococcal disease [1-4]. However, the most compelling reason for their widespread use may lie with indirect protection (herd immunity) that these vaccines provide to the non-vaccinated part of the population [5-7]. This means that a considerable proportion of prevented cases of disease may result from reduced transmission of asymptomatic nasopharyngeal carriage of pneumococci in the population [8]. Such indirect protection is based on the ability of the conjugate vaccines to reduce acquisition of pneumococcal carriage [9,10], a pre-requisite of pneumococcal disease.

Transmission of pneumococcal carriage is particularly efficient among children, both in families and day care facilities [11,12]. A number of studies have attempted to quantify the effect of exposure to pneumococci in terms of surrogate measures, such as family size, number of siblings, crowding, attendance to day care, or weekly hours spent in day care [13-15]. By contrast, only few studies have quantified direct exposure to carriers of pneumococci in a serotype specific manner. The family studies that have recorded carriage in all family members reveal a higher intensity of transmission among family members in comparison to acquisition from the general community [16-19]. Similar results apply to children with close contacts in school classes [20]. In all these studies exposure to pneumococcal carriage had been measured in one mixing group only, the family or the school class.

Enhanced transmission in families and day care facilities implies that transmission in the whole population occurs through micro-epidemics, i.e., temporally and spatially localised outbreaks of carriage in these "mixing" groups. Theoretical analyses have shown that groups with intensive within-group transmission induce an amplifying effect on transmission in the population [21]. In fact, empirical data and simulation models have emphasized the role of day care centres enhancing pneumococcal circulation in the population [15]. The amplifying effect of the mixing groups can be characterised by the average size of the outbreak, where the relevant measure of size is the total number of episodes of carriage during a single micro-epidemic of carriage [cf. [21]]. The outbreak size has bearing on the transmission potential of pneumococcal carriage, which eventually translates to the vaccination effort needed to stop transmission in a population (cf. [22,23]).

The notion of outbreaks of pneumococcal carriage is strengthened by the observation that different pneumococcal serotypes or strains may dominate temporally and locally in different day care facilities [24-26]. It is an interesting question to which extent such patterns in pneumococcal carriage are determined by chance alone in a net of inter-connected clusters (day care groups and families), with different intensities of transmission within day care groups and the general population. Alternatively, the pattern could at least partly reflect intra-species competition between different serotypes.

In this study we report a novel analysis of pneumococcal transmission. The analysis is based on a data set that to our knowledge is the first to measure direct exposure to pneumococci within families and day care facilities in the same study cohort. Based on these data, we have previously shown that exposure to pneumococci both within day care centres and within family are important risk factors for acquisition of carriage in day care attendees [26]. Using statistical modeling, we now quantify the importance of the two mixing groups for pneumococcal transmission on the individual and population levels. We assess the importance of transmission vs. between-serotype competition in producing the observed patterns of carriage. We then treat pneumococcal transmission as outbreaks of carriage, occurring in inter-related groups of day care attendees. Based on this, we derive an estimate of the group-to-group basic reproduction number for a single serotype, describing the amplifying effect of within-group transmission. Finally, we assess the critical vaccine efficacy against acquisition to obtain herd immunity threshold, i.e., to eliminate pneumococcal carriage, and discuss the implications of this for pneumococcal disease.

## Methods

### The empirical data

The data have been described in detail elsewhere [26]. Briefly, all attendees with their family members and the employees in three day care centres (DCCs) in the Tampere area, Finland, were invited to participate in a longitudinal study of pneumococcal carriage. Altogether, 213 individuals consisting of 61 day care attendees (59 index children, and 2 siblings who entered the DCC later during the study), 29 siblings, 86 family members > 18 years of age, and 37 employees, were enrolled as study participants. In the three DCCs, the 25, 18 and 18 attendees belonged to 20, 13 and 12 families, respectively. The mean age of the attendees in the beginning of the study was 4.1 years (range 1.2–6.6). The attendees account for 74%, 26% and 40% of the mean number of children (34, 69, and 45) attending the three DCC's. The term *day care cohort *refers to all individuals connected to a DCC, including the non-participants.

During the follow-up between September 2001 and May 2002, nasopharyngeal (NP) samples of pneumococcal carriage were collected from the study participants at 10 monthly visits. For most of the 213 participants the data are almost complete: 87% of the individuals have 9 or 10 NP samples. In particular, missing data are few in the participating families. Altogether 1941 samples were taken from the individuals. Each sample comprises of the calendar time of sampling, the age of the individual at sampling, pneumococcal carriage (yes/no) and, in case of carriage, the serotype of the isolate. In addition, the contact groups (family, DCC) for each individual are known. Table 1 summarises the data as episodes of carriage. The reported numbers of participants that had antibiotic treatment during the month preceding the sample was 54 (9.6% of the samples) in day care attendees, 25 (8.9%) in siblings, 38 (4.8%) in family members > 18 years of age, and 26 (7.5%) in day care employees.

**Table 1:** Numbers of episodes of pneumococcal carriage in the three day care cohorts for day care attendees and for all participants.

	Day care attendees (N = 61)			All participants (N = 213)		
**Serotype**	**DCC1**	**DCC2**	**DCC3**	**DCC1**	**DCC2**	**DCC3**

9V	15	0	0	21	0	0
18C	0	0	13	0	0	20
3	9	2	6	11	2	6
19F	0	8	0	1	14	1
15B/C	2	4	5	3	6	6
11A	1	2	3	1	4	7
19A	7	0	0	8	0	0
35F	3	2	0	3	4	1
14	0	2	2	0	4	3
6B	1	1	0	1	4	1
22	0	2	0	0	4	0
33	2	0	0	3	0	0
38	2	0	0	2	0	0
6A	1	1	0	1	1	0
9N	0	0	1	0	1	1
10	1	0	0	1	0	0
16	1	0	0	1	0	0
18B	0	1	0	0	1	0
35B	0	0	1	0	0	1
7	0	0	1	0	0	1
Non-typables	0	0	0	0	3	3

**Total**	**45**	**23**	**30**	**57**	**48**	**51**

Clustering of carriage is most evident for serotypes 9V, 18C, and 19A, each being the dominant type in one DCC and not detected in the two other DCCs. Serotype specific episodes of pneumococcal carriage were constructed from the observed data by combining consecutive samples of the same serotype into episodes.

### Statistical methods

#### Notations and Definitions

The time of origin *t*_min _is defined as the day before the first NP sample in the data was taken. At any time *t*, the state of individual *i *is one of the *n*_*s *_+ 1 possible *states*, i.e., the individual is either a carrier of one of *n*_*s *_serotypes, *s*_*i*_(*t*) ∈ {1,...,*n*_*s*_}, or is a non-carrier, *s*_*i*_(*t*) = 0. The process , *r*,*s *= 1,...,*n*_*s*_, *r *≠ *s*, counts the number of times the individual has moved from state *r *to state *s *since *t*_min _by time *t*. Further, for a study cohort of *n *individuals the history of the *n *× (*n*_*s *_+ 1) × *n*_*s *_counting processes  at time *t *is denoted by *H*_*t*_.

#### Transmission model

Acquisition and clearance of pneumococcal serotypes is modelled through the following stochastic intensities for processes :(1)

where  is the indicator of individual *i *being in state *r *at time *t*. The model considers two age groups; adults (age *a*(*i*) ≤ 7 years) and children (*a*(*i*) < 7). The rate for individual *i *is given by

Here  is the baseline rate of acquisition of serotype *s *in a non-carrying child. To account for possible competition between pneumococcal serotypes in colonising the host, a competition parameter *φ *≥ 0 is used to scale the rate of acquisition rate in an individual already carrying another serotype. For a carrying child, *t*_*acq *_is the acquisition time of the ongoing episode of carriage, and *α*^0^(*t *- *t*_*acq*_) = *ρμ*(*μ*(*t *- *t*_*acq*_))^*ρ*-1^, for *ρ *= 3, is the clearance rate (the intensity function of a Weibull distribution) at time *t *- *t*_*acq *_after acquisition. To account for differences between children and adults the acquisition rates are multiplied with *η*_*a*(*i*) _= *η*1(*a*(*i*) ≥ 7) + 1(*a*(*i*) < 7), where *η *is the relative acquisition rate in adults versus children. Similarly, the clearance rate is multiplied with *δ*_*a*(*i*) _= *δ*1(*a*(*i*) ≥ 7) + 1(*a*(*i*) < 7), where *δ *is the relative clearance rate in adults versus children.

In addition to including a community force of infection *κ*, the baseline rate of acquisition in individual *i *at time *t *takes into account serotype specific exposure within the two mixing groups (family of size  and DCC of size ):

Thus, the baseline rate depends on the number of carriers of serotype *s *in the individual's family () and day care centre () at time *t*. Here  is the rate at which a family member carrying serotype *s *transmits carriage to a non-carrying susceptible family member (similarly for the rate *β*^*dcc *^within the DCC). The second (DCC) term is included in the acquisition rates of the DCC attendees and employees only.

#### Hierarchical model

Making likelihood-based inference about the model parameter *θ *= {*β*^*fam*^, *β*^*dcc*^, *κ*, *μ*, *φ*, *η*, *δ*} requires knowing the exact event times and types (i.e. specific transitions). However, the data consist of monthly samples only. The problem was tackled by adopting a Bayesian latent process approach, where using a Markov chain Monte Carlo (MCMC) algorithm the space of possible carriage histories *H*_*t*_, i.e., that of latent processes consistent with the observed data, was effectively sampled to produce estimates of the unknown parameters (see Appendix) [16,20]. Parameter estimates are given in terms of their posterior means and 90% credibility intervals (90% CI).

#### Posterior predictive model validation and description of outbreaks

Transmission of carriage of 13 serotypes in a single day care cohort was simulated, based on model (1) and samples from the posterior distribution of the model parameters. The simulated day care cohort consisted of 50 DCC attendees (children) and their family members (13 families containing two day care attendees and two adults, and 24 families containing one day care attendee and three adults). The posterior predictive simulations were used

A. to validate the model by comparing the posterior prediction of the serotype distribution to the actually observed distribution,

B. to quantify transmission patterns, i.e., who infected whom within the cohort, by monitoring exposure at each acquisition, and

C. to characterise outbreaks of pneumococcal carriage by monitoring individual outbreaks for the total number of episodes during one outbreak and the duration of outbreak.

After a sufficient burn-in period of 1000 days, carriage processes for cases A and B were followed for 270 days, and until the end of outbreak for case C. An outbreak was defined as the time interval from the first acquisition of a specific serotype in the cohort until its (temporary) disappearance from the cohort. In case A the model validation was based on 10 evenly spaced samples gathered from each day care attendee. To account for possible bias in the observed serotype distribution, due to incomplete sampling (i.e. missing data), the validation was repeated based on a sub-sample of 20 DCC attendees. In case B, the total number of acquisitions was divided into those from the day care centre, the family, or the community in proportions of exposure from the three sources. The results are based on 1000 posterior predictive simulations.

#### Transmission potential and the critical vaccine efficacy

For any given serotype, the potential of within-group outbreaks to sustain transmission in the whole population is characterized by the average number of infectious contacts emanating from a single outbreak [21], the so called group-to-group reproduction number R*. In the present context, this value can be approximated by R* = *λsD*, where *λ *is the serotype specific rate at which day care attendees infect other day care attendees in the community, *D *is the mean duration of a carriage episode in a day care attendee, and *s *is the average number of carriage episodes in day care attendees in an outbreak originating from a single carrying DCC attendee in a initially susceptible (i.e. non-carrying) day care centre in the absence of competing serotypes, and in the absence of community force of infection.

An approximation to rate *λ *can be inferred from the stationary prevalence of day care attendees in the community, and the pneumococcal acquisition rate from the community (*κ*), assuming homogeneous mixing on the population level. Specifically, if the prevalence of carriage in day care attendees is *p *(~0.25), the serotype specific community rate can be expressed as *λ *= 13 *κ*/*p*. Posterior predictive simulations based on a day care cohort of size 50 (with a similar structure as above) were used to estimate *s*. The simulations were performed by colonising a random child in an initially susceptible cohort and then recording the size of the outbreak. In total 5000 simulations were performed.

Let *ν *denote the vaccine efficacy, i.e., the percentage reduction in the rate of acquisition for a specific serotype. The effective post-vaccination group-to-group reproduction number is then given by , where *s*_*v *_is corresponding average number of carriage episodes under vaccination. The critical efficacy *ν*_*c *_is the solution to the nonlinear equation . By applying the idea of proliferation of carrying individuals (day care attendees) in a community of day care centres (cf. [27]), one can infer that the individual-to-individual basic reproduction number for a pneumococcal serotype is give by (1 - *ν*_*c*_)^-1^.

## Results

### Prevalence of pneumococcal carriage

The 90% interval of the posterior predictive prevalence of pneumococcal carriage in children in a DCC of size 50, calculated from 10 monthly samples was [21%, 46%] with mean value 33%. For a sub-sample of 20 DCC attendees the corresponding interval was [21%, 48%], which is in line with the observed prevalence in the three centres (27%, 26%, and 23%). Further, in the sub-sample of 20 DCC attendees on a single sampling round the 90% interval for the posterior predictive prevalence was [10%, 60%], also comparing well with the observed prevalence that varied between 9% and 56%.

### Community acquisition and the effect of competition

The posterior mean rate of acquisition from the community (*κ*) was 0.0059 per month per serotype in a non-carrying child (90% CI [0.0043, 0.0078])(Table 2). The posterior mean of the competition parameter (*θ*) was 0.68 (90% CI [0.35, 1.10]), indicating reduced ability of new strains to occupy an already colonised nasopharynx.

**Table 2:** Estimates of the model parameters.

Model parameter	Posterior mean	5% quantile	95% quantile
Community acquisition rate κ (per month)	0.0059	0.0043	0.0077

Family transmission rate *β*^*fam*^ (per month)	0.36	0.23	0.52

DCC transmission rate *β*^*dcc*^ (per month)	0.53	0.38	0.71

Clearance rate *μ* (per month)	0.69	0.64	0.75

Competition parameter *θ*	0.68	0.35	1.10

Relative susceptibility *η* (adults vs. children)	0.41	0.28	0.58

Relative clearance rate *δ* (adults vs. children)	1.23	1.06	1.41

The table presents the mean as well as the 5 and 95 percent quantiles of the marginal posterior distributions of the 7 model parameters

### Within-family and within day care acquisition

The posterior mean within-family transmission rate (*β*^*fam*^) in children (age < 7 years) was 0.37 per month (90% CI [0.23 0.52]). Adults (≥ 7 years) were less susceptible to acquisition, with a relative transmission rate of 0.41 (90% CI [0.28 0.58]). For a carrying child, it thus takes 4.5 months on average to infect another family member in a family of one susceptible (child) sibling and two susceptible adults. The within-DCC transmission rate (*β*^*dcc*^) was similar to the within-family rate, with posterior mean 0.53 per month (90% CI [0.38, 0.71]). However, because the group size is larger than in the family, this corresponds to an average of only 1.9 months for a carrying child to infect another child in a totally susceptible DCC.

According to posterior predictive simulations of the transmission model in a DCC of size 50, 65% of acquisitions in day care attendees were from fellow day care attendees, 25% from the community, and only 10% from family members. In introduction of a new serotype into the family, in 82% of the cases the introductory individual was a day care attendee, and in 71% of these instances the acquisition had been from a fellow DCC attendee.

### Clearance of carriage

The posterior mean rate (*μ*) of clearing pneumococcal carriage was 0.69 per month (90% CI [0.64, 0.75]). Clearance in adults (≥ 7 years of age) occurred faster, with the mean relative rate of 1.23 in comparison to children (90% CI [1.06, 1.42]). In the absence of competing acquisition from other serotypes, the posterior predictive mean duration of carriage in children would thus be 39 days in contrast to only 32 days in adults. In the posterior predictive simulations the mean length of a single episode for children was 33 days (95%CI [5, 50]), 6 days less than the implied mean duration in the absence of competition.

### Model validation

**Figure 1:**
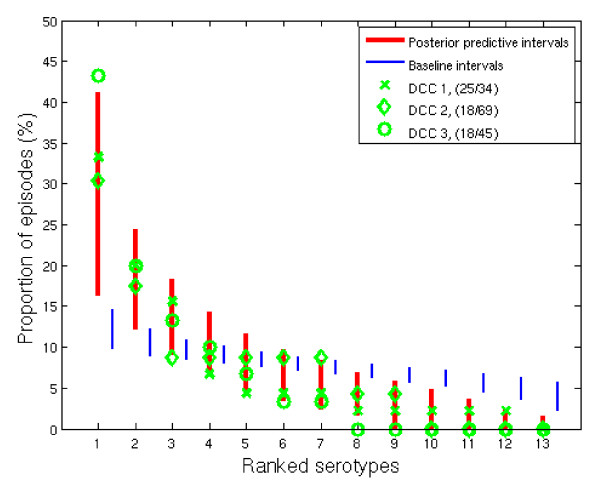
**Model validation**. The cross, diamond, and circle present the observed proportions in the attendees in the three day care centres. The proportions are ranked in ascending order. The thick lines denote the 90% posterior predictive intervals of the ranked proportions, calculated from a sub-sample of 20 day care attendees. The narrow lines are based on episodes with random serotypes, showing the "baseline" distribution that results from ranking only. The posterior predictions were based on 1000 simulations of a day care centre cohort consisting of 50 day care attendees and their family members. The size of the three DCCs as the number of day care attendees together with the number of participating attendees is given in the parenthesis.

Figure 1 presents posterior predictions of the frequency distribution of serotypes in the day care attendees of a single day care cohort, together with the actually observed data from the three cohorts. The serotypes are ranked according to their prevalence. The predicted and observed distributions are similar, showing that our model was successful in producing the observed pattern. Specifically, the highly skewed distribution is a consequence of serotype-specific clustering. Of note, this clustering is produced by within-cohort transmission and between-serotype competition even under the assumed exchangeability of serotypes in terms of their rates of acquisition and clearance. To distinguish the pattern from clustering produced simply by ranking the serotypes, a baseline distribution is shown. The baseline was constructed by assigning random serotypes to each episode and then ranking them.

### Characteristics of outbreaks

The obvious clustering in the data, reproduced by the model predictions, indicates that individual serotypes cause outbreaks of carriage within day care cohorts. The posterior predictive mean duration of such outbreaks was 2.8 months, with the inter-quartile range [0.9, 3.5]. The mean number of serotypes carried in a day care cohort at any time was 7.5, which means that on average 2.7 outbreaks (new serotypes) are introduced into the cohort each month. Epidemiologically, a key characteristic of an outbreak is its size, i.e., the total number of episodes during the outbreak [28]. According to the data and our model, the posterior predictive size of the outbreak has a skewed, heavy-tailed distribution: 39% of outbreaks were of size 1 and the mean size was 7.6 episodes, consisting of 5.7 episodes in the day care attendees and 2.0 episodes in the adult family members. The size of the outbreak depended on the initial carrier. If the initial carrier was a child, the average size was 9.6 (7.8 + 1.8), whereas if the initial carries was an adult, the size was only 5.3 (3.2 + 2.1).

### Transmission potential

**Figure 2:**
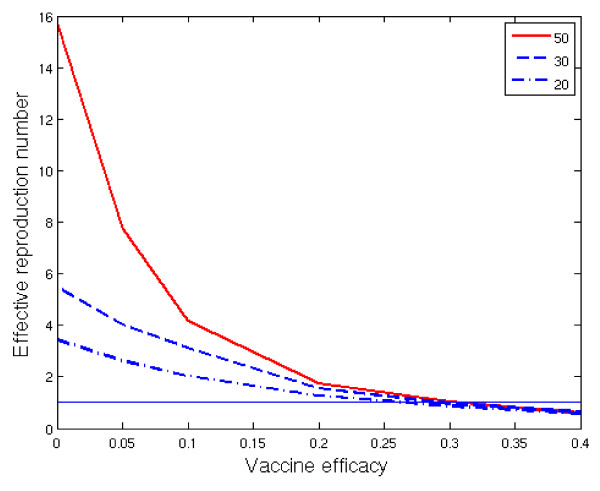
**The critical vaccine efficacy**. The group-to-group reproduction number  under vaccination in a population of day care cohorts of equal size, for different values of vaccine efficacy against acquisition. For each line the size of the day care cohort is given as the number of day care attendees (50, 30, and 20). The entire cohort including family members were used in the simulations. In each simulation roughly half of the day care attendees had a sibling attending the same day care centre, i.e., one third of the families had two children in the day care centre and two thirds of the families had one child in the day care centre.

From posterior predictive simulations, the size of the outbreak in the absence of competition was 46.6 (39.5 + 7.1), if the initial carrier was a child. The group-to-group reproduction number R* (based on children only) calculated from posterior predictive simulations was 15.8, with a 90% posterior probability to be less than 25. The critical vaccine efficacy, searched for by simulations, was found to be 0.3 (Figure 2) and the corresponding child-to-child reproduction number, inferred via the critical vaccine efficacy, was 1.4.

## Discussion

We analysed longitudinal data on pneumococcal carriage in three cohorts of day care children and their family members. Rates of pneumococcal acquisition, conditional on serotype specific exposure, were estimated within a Bayesian framework, utilising latent processes of carriage in continuous time. To adjust for missing data, unobserved events of acquisition and clearance were augmented statistically. The results show that pneumococcal carriage occurs as serotype-specific micro-epidemics, i.e., as outbreaks of carriage among day care attendees and their family members. Transmission within day care centres is the driving force of pneumococcal transmission in a population. In particular, outbreaks of pneumococcal carriage in day care centres cause an amplifying effect that contributes in maintaining circulation of pneumococci in the population. For a single pneumococcal serotype, the group-to-group reproduction number was estimated at 16, the individual-to-individual reproduction number at 1.4, and the critical vaccine efficacy against carriage at 0.3.

Although the conditional transmission rates within families and within day care centres were similar, the role of families in creating and maintaining micro-epidemics is minimal. This is due to the smaller size of families in comparison to day care centres and to the significantly lower susceptibility of adults compared to children. Also, as indicated by the predictive simulations, the day care attendees were the dominant source in introduction of new pneumococcal serotypes into the family. This is in line with [29], where pneumococcal carriage in DCC attendees was shown to associate with carriage in their younger siblings.

One of the goals of the present study was to assess the relative importance of transmission and between-serotype competition in shaping the clustered pattern of carriage. We showed that both intense within day care transmission and competition are needed. In particular, model simulations showed that mere transmission in the absence of competition produces too large outbreaks (mean outbreak size 46.6, in comparison to 9.6 in the presence of competing serotypes). The role of competition in our model was thus to limit the size of outbreaks through reduced duration of carriage, due to acquisition of other serotypes.

Serotype-specific clustering of pneumococcal carriage within day care cohorts implies that contact rates are larger among individuals within the same cohort than between individuals from different cohorts. Pneumococcal transmission can then be viewed as occurring among a community of day care centres in terms of the idea of group-to-group transmission. In such a set-up, [27] derived threshold parameters for eliminating endemic circulation of a highly infectious agent by considering proliferation of infected individuals or households. In [21] an analogous threshold theorem was derived for an infection that does not confer immunity against re-infection (cf. pneumococcal carriage). We applied the latter approach to determine the basic reproduction number for the proliferation of day care centres that carry a specific serotype. Assuming a homogeneous size of the DCC (N = 50) we found that R* was 16.

We then asked what is the critical efficacy against pneumococcal acquisition for elimination of a specific serotype, if all day care attendees were to be vaccinated. The critical vaccine efficacy *ν*_*c *_was found to be 0.3, which means that although the serotype specific R* appears large, vaccination works very effectively in a clustered set-up. The explanation is that in addition to reducing susceptibility to pneumococcal carriage in individuals, vaccination induces a herd effect on transmission within DCCs, thus creating a counter-effect to the amplifying effect of the cohort. As shown in Figure 2, the critical vaccine efficacy is robust to the size of the DCC. Figure 2 can also be used to assess the dependence of the critical vaccine efficacy on the estimate of rate *λ *(0.31 per month), at which a child transmits carriage to outside its own day care cohort. Specifically, if this rate is an over-estimate, e.g. half of the actual value, the critical vaccine efficacy is read from the intersection with line R* = 0.5, implying somewhat higher values for the critical efficacy. If *λ *is an under-estimate, the critical efficacy would be lower than 0.3, with some heterogeneity according to the assumed group size.

The individual-to-individual basic reproduction number for a pneumococcal serotype was estimated as 1.4. This low reproduction number corresponds well to an SIS type of infection (pneumococcal carriage) [30,31], for which the required vaccination effort is typically of the order of the prevalence of infection. Thus, for a typical serotype with prevalence of the order of 10% at maximum, the number is actually quite large, describing the transmission potential in the absence of competition by other serotypes. As the individual-to-individual basic reproduction number is a direct function of the critical vaccine efficacy, its assessment is also robust to the size of the DCC. In addition, competition by non-vaccine serotypes would induce a beneficial effect, thus implying an even smaller critical efficacy against carriage.

The observed serotype distribution was slightly more clustered than that produced by posterior predictive estimates. A possible explanation is the existence of sub-groups, classes, within each day care centre, which was not considered in the model. DCC1 and DCC3 consisted of two classes and DCC2 of three classes. The data for some serotypes suggest that transmission within the sub-groups is higher than between the sub-groups, although this was not consistent over serotypes and day care centres (data not shown). Another explanation for the even stronger clustering in the observed data is the simplifying assumption of a constant community exposure over the follow-up time period. It is likely that community exposure to the included serotypes was temporally heterogeneous, thus increasing clustering.

Within each day care cohort the analysis considered 13 serotypes only, which was the maximum number of serotypes found in a single cohort. We experimented also with a model were all 21 serotypes found in the present study were taken into account in each cohort. Due to the assumption of equal rates, the overall community force of infection divides equally between all serotypes, thus resulting in a smaller community acquisition rate per serotype. However, the model validation with revealed a poor fit for this model, with too many serotypes present and too little clustering.

Only 21 out of the 91 serotypes were observed during the follow-up. Especially there were no isolates of serotype 23F, which was one of the most prevalent serotypes in a contemporaneous study from the same geographical area. Due to the high transmission rate within the day care cohorts, samples within a cohort are highly correlated. Thus the effective sample size to determine the serotype distribution is much smaller than the number of samples. However, we hypothesise that by sampling a large number of day care cohorts we would have encountered micro-epidemics of other serotypes and the overall serotype distribution would have resembled that of the population.

The assumption of identical parameter values for all serotypes goes against the general understanding that serotypes have different transmission properties and is thus a possible limitation of the study. The main reason to treat all serotypes as identical in the present study is that this approach significantly reduces the number of parameters, while still allowing for 1) comparisons between within-family and within day care exposure, 2) the inspection of age dependency in susceptibility of acquisition and duration or carriage, and 3) quantification of competition between serotypes. However, in the interpretation of the results one should keep in mind that the results represent the average behaviour of the observed serotypes and in reality there may be differences that our model is not able to address. Obviously, this could apply also to serotype 23F and other usually carried serotypes, not prevalent in the sample of the present study, although we do not consider this likely.

The statistical analysis in this study that did not take into account the non-participants resulted in a higher community acquisition parameter (mean value 0.0082 in comparison to 0.0059). This reflects the fact that including more people into the cohort allows acquiring the same amount of acquisition from outside of the cohort with a smaller community acquisition value. A similar bias was evident in an approach where the non-participant DCC attendees were included into the analysis but their family members were left out. Also in this approach during the MCMC estimation the prevalence of pneumococcal carriage in the non-participant DCC attendees was consistently lower than that of the participating DCC attendees (calculated from the latent processes). The reason is that the non-participant DCC attendees where not exposed by their families, which led to underestimation of the true level of exposure.

Our model did not take into account all factors that are known to affect pneumococcal carriage. For example, respiratory infections are known to be associated with increased acquisition of carriage and antibiotic treatment temporarily reduces carriage. However, the aim of the current analysis was to describe natural pneumococcal transmission in young children, for which exposure to pneumococci is by far the most important "risk factor" [17]. The results of our analysis describe the micro-epidemic pattern of carriage in a population with a relatively low use of antibiotics.

The efficacy of the pneumococcal conjugate vaccines against most vaccine types (serotypes included in the vaccines) has been estimated to be about 0.5 (e.g. [9,32]), which surpasses the critical vaccine efficacy (0.3) inferred in our study. We therefore conclude that in the present setting conjugate vaccines would be efficacious enough to eliminate carriage of at least most vaccine serotypes. Further, as carriage is a pre-requisite of pneumococcal disease, our results predict that herd protection, provided by elimination of transmission, is on its own sufficient to eliminate the majority of pneumococcal disease caused by the vaccine serotypes.

Our results describe the dynamics of natural carriage in day care cohorts in Finland. The results as such may not be directly applicable to countries with different epidemiology of pneumococccal carriage, i.e., with higher prevalence carriage. However, similar models based on the community structure can be used to assess the importance of group-to-group transmission on pneumococcal carriage and its elimination.

## Conclusion

Both within-DCC transmission and between-serotype competition play an important role in shaping micro-epidemic transmission of pneumococcal serotypes. The birth and expansion of outbreaks of carriage within day care cohorts are enabled by the intense within-group transmission. Competition by other serotypes restricts the size of outbreaks. The amplifying effect of day care cohorts, characterised by the mean size of an outbreak, promotes the spread of pneumococcal serotypes within a population. Although the size of the DCC has a large effect on the reproduction number, its impact on the critical vaccine efficacy is small. In a population of DCCs, the vaccine efficacy against acquisition of carriage, needed to eliminate transmission of an individual serotype in the absence of competing serotypes, was estimated as 0.3 only, which will translate to a strong herd protection against pneumococcal disease.

## Competing interests

The authors declare that they have no competing interests.

## Authors' contributions

FH participated in the design of the statistical analysis and in writing the manuscript, and performed the statistical analysis. PE participated in the development of the statistical analysis and in writing the manuscript. TL participated in designing the study and writing the manuscript. KA participated in designing the study, in the statistical analysis, and in writing the manuscript. All authors have read and approved the final manuscript.

## Appendix

### The complete data likelihood function

For individual *i*, denote by  the set of times the carriage status changes from state *r *to state *s *in the time interval ]*t*_min_, *t*_max_], where *t*_max _is the day after the last NP sample in the data is taken. Let *T*_*i *_= {, *r*, *s *= 1,...,*n*_*s*_} be the collection of all times individual *i *changes carriage status. The likelihood function for *n *individuals on the time interval ]*t*_min_, *t*_max_] defined by model (1) is(2)

where the unknown model parameters are gathered into the vector *θ *= {*β*^*fam*^, *β*^*dcc*^, *κ*, *μ*, *φ*, *η*, *δ*} [33]. Within each day care cohort the transmission of 13 serotypes (*n*_*s *_= 13) was considered, which was the maximum number of serotypes observed in one day care cohort during the follow-up.

### Prior distributions

The prior distribution of the community acquisition rate *κ*, the within-family transmission rate *β*^*fam*^, the within-DCC transmission rate *β*^*dcc*^, and the clearance parameter *μ *were assigned a normal distribution with mean zero and standard deviation 3000 (rate per month), constrained to positive values only. The relative acquisition rate *η *and the relative clearance rate *δ *were both assigned a uniform prior on the interval [0,10]. For the competition parameter *φ *we assumed a prior proportional to *φ*^-1^, reflecting equal prior probabilities for the events *φ *< 1 and *φ *< 1. In addition the probability to carry a serotype at the beginning of the follow up was fixed to 0.25 for the day care attendees and to 0.10 for the others. The maximum number of carriage episodes per individual was set to 10.

### The non-participants

To ensure the correct contact structure and level of exposure, the non-participating members of the day care cohort were included in the statistical analysis. These included the ten family members of participating day care attendees that had no observations during the follow-up. In addition, carriage histories were augmented for 77 non-participating day care attendees and their family members. The family size of the non-participating day care individuals was assumed to be four according to the mean reported family size. Also in line with the observed data, half of the non-participating day care attendees were assumed to have a sibling in the same day care. This resulted in augmenting 9(7), 51(38), and 27(20) day care attendees (families) in DCC1, DCC2, and DCC3, respectively. Thus the analyses are based on 213 participants (at least one NP sample) and 270 non-participants (no NP samples).

Since no measurements were available from the non-participating individuals, their carriage histories relied solely on the model parameters and on the carriage histories of the participating members. Therefore, in the parameter estimation step of the Markov chain Monte Carlo (MCMC) algorithm an *ad hoc *approach (cf. the "cut" function in WinBUGS User Manual [34]) was adopted, where the information flow from the non-participants was discarded, i.e., the likelihood function (2) was calculated as a product over the participants only. The non-participants were taken into account in determining exposure to pneumococci in the participants.

### The Markov chain Monte Carlo (MCMC) algorithm

The MCMC algorithm used to produce estimates of the model parameters was tailor-made using Matlab (version 7.5). The sketch of the MCMC algorithm is as follows:

1. Initialise model parameters *θ*

2. Initialise latent processes

3. Update parameters *θ*

a. one at a time, update *κ*, *β *and *μ*

b. update *η *and *δ *as a block

4. Update latent processes for a random sample of 20% of the individuals

a. propose a new episode as described below → accept/reject proposal

b. propose moving an event time → accecpt/reject proposal

5. Iterate steps 3 and 4 for a predefined number of rounds

In total the MCMC algorithm was run for 15000 rounds after 5000 burn-in rounds.

### Updating the latent processes: proposing a new episode

In the MCMC algorithm for each individual we first initialise a path consistent with the observed panel data. The path *H*_*i *_of individual *i *consists of the carriage status at time *t*_0 _and a series of events times, acquisition/clearance times *T*_*i*_, together with the corresponding event types.

At each MCMC round the path of a chosen individual is updated by the following algorithm.

1. Choose randomly one of the sampling intervals [*S*_*v*_, *S*_*v*+1_], *v *= 1,...,(*N *- 1), where *S*_1 _and *S*_*N *_are the beginning and the end of the follow-up, and *S*_2_,...,*S*_*N*-1 _are the individuals sampling times in ascending order.

2. Within the chosen sampling interval choose randomly an episode [*t*_*k*_, *t*_*k*+1_], *k *= 1,...,(*M *- 1), where *t*_1 _= *S*_*v *_and *t*_*N *_= *S*_*v*+1 _are the beginning and the end of the interval, and *t*_2_,...,*t*_*M*-1 _are the individual's acquisition/clearance times within the interval in ascending order.

3. Define conjoin probabilities *P*_*left *_and *P*_*right *_(needed later)

a. if *S*_*v *_= *t*_*k*_, then *P*_*left *_= 0, otherwise *P*_*left *_= 0.5

b. if *S*_*v*+1 _= *t*_*k*+1_, then *P*_*right *_= 0, otherwise *P*_*right *_= 0.5

4. Propose limits [, ] for a new episode

a. with probability (1 - *P*_*left*_)(1-P_right_), pick randomly , ∈ [*t*_*k*_, *t*_*k*+1_], so that  <

b. with probability *P*_*left*_*P*_*right*_,  = *t*_*k *_and  = *t*_*k*+1_

c. with probability (*P*_*left*_)(1 - *P*_*right*_),  = *t*_*k *_and pick randomly  ∈ ]*t*_*k*_, *t*_*k*+1_]

d. with probability (1 - *P*_*left*_)(*P*_*right*_), pick randomly  ∈ [*t*_*k*_, *t*_*k*+1_[ and  = *t*_*k*+1_

5. Propose a "sero"type for the new episode

a. if episode [*t*, *t*_*k*+1_] is non-carriage episode, propose a serotype randomly from the *n*_*s *_possibilities

b. if episode [*t*_*t*_, *t*_*k*+1_] is carriage of one of the serotypes, propose a non-carriage episode

6. Merge similar types

a. if  = *t*_*k*_, and the "sero"type of the proposed episode and the previous episode are the same, merge the episodes, i.e.,  = *t*_*k*-1_

b. if  = *t*_*k*+1_, and the "sero"type of the proposed episode and the following episode are the same, merge the episodes, i.e.,  = *t*_*k*+2_

7. In order to calculate the probabilities of the back-proposal we define  and 

a. if *S*_*v *_= ,  = 0, else  = 0.5

b. if *S*_*v*+1 _= ,  = 0, else  = 0.5

8. Accept the proposed episode with probability

Where *H*_*i *_and  are the present and the proposed path (history) for individual *i*, *M*_*i *_is the observed data for individual *i*, *P *is the prior of the complete data (the likelihood function of the model parameters), *P*_*c *_is the likelihood function of the complete data (is one if the complete data is consistent with the observed data and is zero otherwise), and *Q*(*u*|*v*) is the probability of proposing path *u *given path *v*. The exact form of *Q *can be derived from steps 1 to 7. For example, if we propose to add a carriage episode [, ] within a non carriage episode [*t*_*k*_, *t*_*k*+1_], where *t*_*k *_< < <*t*_*k*+1_, the proposal probability is

and the back proposal probability is

## Pre-publication history

The pre-publication history for this paper can be accessed here:

http://www.biomedcentral.com/1471-2334/9/102/prepub
